# Ca14-3-3 Interacts With CaWRKY58 to Positively Modulate Pepper Response to Low-Phosphorus Starvation

**DOI:** 10.3389/fpls.2020.607878

**Published:** 2021-01-14

**Authors:** Jinsen Cai, Weiwei Cai, Xueying Huang, Sheng Yang, Jiayu Wen, Xiaoqin Xia, Feng Yang, Yuanyuan Shi, Deyi Guan, Shuilin He

**Affiliations:** ^1^National Education Ministry Key Laboratory of Plant Genetic Improvement and Comprehensive Utilization, Fujian Agriculture and Forestry University, Fuzhou, China; ^2^Key Laboratory of Applied Genetics of Universities in Fujian Province, Fujian Agriculture and Forestry University, Fuzhou, China; ^3^Agricultural College, Fujian Agriculture and Forestry University, Fuzhou, China

**Keywords:** Capsicum annuum, CaWRKY58, Ca14-3-3, CaPHR1, phosphorus deficiency

## Abstract

Low-phosphorus stress (LPS) and pathogen attack are two important stresses frequently experienced by plants in their natural habitats, but how plant respond to them coordinately remains under-investigated. Here, we demonstrate that CaWRKY58, a known negative regulator of the pepper (*Capsicum annuum*) response to attack by *Ralstonia solanacearum*, is upregulated by LPS. Virus-induced gene silencing (VIGS) and overexpression of CaWRKY58 in *Nicotiana benthamiana* plants in combination with chromatin immunoprecipitation (ChIP) and electrophoretic mobility shift assays (EMSA) demonstrated that CaWRKY58 positively regulates the response of pepper to LPS by directly targeting and regulating genes related to phosphorus-deficiency tolerance, including PHOSPHATE STARVATION RESPONSE1 (PHR1). Yeast two-hybrid assays revealed that CaWRKY58 interacts with a 14-3-3 protein (Ca14-3-3); this interaction was confirmed by pull-down, bimolecular fluorescence complementation (BiFC), and microscale thermophoresis (MST) assays. The interaction between Ca14-3-3 and CaWRKY58 enhanced the activation of PHR1 expression by CaWRKY58, but did not affect the expression of the immunity-related genes *CaNPR1* and *CaDEF1*, which are negatively regulated by CaWRKY58 in pepper upon *Ralstonia solanacearum* inoculation. Collectively, our data indicate that CaWRKY58 negatively regulates immunity against *Ralstonia solanacearum*, but positively regulates tolerance to LPS and that Ca14-3-3 transcriptionally activates CaWRKY58 in response to LPS.

## Introduction

Plants are confronted with fluctuating ecological environments and are often exposed to biotic or abiotic stresses. Therefore, they have evolved sophisticated defense mechanisms to perceive stress, and to initiate and translate signaling pathways into appropriate defense responses that involve massive transcriptional reprogramming *via* transcription factors (Moore et al., 2011). To maximize fitness, plant growth, development, and responses to environmental cues must be tightly and coordinately regulated, which requires extensive crosstalk among plant responses to stresses, growth, and development (Fujita et al., 2006; Xia et al., 2015; Li et al., 2020). A single transcription factor is often involved in regulating several apparently disparate processes (Rushton et al., 2010), but the molecular details underlying this coordination often remain poorly understood.

Low-phosphorus stress (LPS) is one of the most important abiotic stresses experienced by plants, due to the requirement of phosphorus (P) for plant growth and development, but its limited availability in natural soils (Shen et al., 2011). Under this selection pressure, plants have evolved strategies to maximize its availability and to adapt to LPS. Massive transcriptional reprogramming activated by LPS has frequently been observed in plant species (Lan et al., 2013; Oono et al., 2013; O’Rourke et al., 2013; Fan et al., 2014; Deng et al., 2018; Xue et al., 2018). Many genes are transcriptionally activated by LPS, leading to enhanced P acquisition and utilization; these include genes that encode proteins such as Pi transporters (Miao et al., 2009; Remy et al., 2012; Ayadi et al., 2015; Liu et al., 2018a; Zhang et al., 2019), H^+^-ATPase (Yuan et al., 2017), phosphate transporter PHT4;6 (Hassler et al., 2012), PHOSPHATE STARVATION RESPONSE 1 (PHR1; Motte and Beeckman, 2017; Wang et al., 2019), protein kinases (Lan et al., 2013; Lei et al., 2014), purple acid phosphatases (Lan et al., 2013; Liu et al., 2018c), SPX (Zhang et al., 2016; Liu et al., 2018b; Osorio et al., 2019), and PHOSPHATE1 (PHO1; Hamburger et al., 2002). The processes involved in LPS responses include alterations in root architecture (Jain et al., 2007; Peret et al., 2011, 2014; Lopez-Arredondo et al., 2014; Postma et al., 2014; Strock et al., 2018), modification of the soil chemistry surrounding roots (Lopez-Arredondo et al., 2014) and the activation of metabolism to efficiently use phosphorus (Plaxton and Tran, 2011; Lopez-Arredondo et al., 2014). The transcription of many transcription factor genes is altered in response to LPS; these transcription factors act as positive or negative modulators of the response and include members of the MYB (Khan et al., 2014; Zhou et al., 2017; Wang et al., 2019), WRKY (Wang et al., 2014; Su et al., 2015; Dai et al., 2016), JAZ (Jasmonate-ZIM domain) (Aparicio-Fabre et al., 2013), bHLH (Valdes-Lopez and Hernandez, 2008b), zinc-finger (Devaiah et al., 2007b; Ding et al., 2016), AP2/ERF (Ramaiah et al., 2014), CCAAT box-binding (NF-Y; Qu et al., 2015), and auxin response factor (ARF; Shen et al., 2013) families, as well as PHR1 (Motte and Beeckman, 2017; Wang et al., 2019). Some of these transcription factors might be modulated in a context-dependent manner by interacting with other regulators, such as SPX domain-containing proteins (Zhong et al., 2018) and WRKY proteins (Zhou et al., 2017). In addition, the response of plants to phosphorus deficiency is closely related to other biological processes, such as the response to NO_3_^−^ (Medici et al., 2015), Fe starvation (Dai et al., 2016), cold stress (Dai et al., 2016), S, Fe, Zn (Briat et al., 2015), K (Wang et al., 2002) and in particular, to plant immunity (Hiruma et al., 2016; Luo et al., 2019). The silencing of *TaPT29-6A*, a Pi transporter that plays a major role in Pi uptake from soil by roots, significantly increased the susceptibility of wheat plants to biotrophic, hemi-biotrophic, and necrotrophic pathogens (Zhang et al., 2019). Furthermore, the levels of secondary metabolites involved in plant immune reactions, including benzoxazinoids and flavonoids, differed significantly in plants grown under Pi-deficient conditions (Luo et al., 2019), indicating extensive crosstalk between plant response to phosphorus-deficiency stress and pathogen attack. However, the underlying mechanism for this crosstalk remains largely uncharacterized.

The WRKY proteins constitute one of the largest transcription factor families in many species (Eulgem et al., 2000), and WRKY family members have been implicated in diverse biological processes and particularly in plant immunity, by specifically targeting the conserved cognate W-box within the promoter regions of their target genes (Eulgem, 2006; Eulgem and Somssich, 2007; Pandey and Somssich, 2009; Ishihama and Yoshioka, 2012; Buscaill and Rivas, 2014; Birkenbihl et al., 2017). WRKY transcription factors, including AtWRKY6 (Bakshi et al., 2015; Decker et al., 2017; Ye et al., 2018), WRKY42 (Sunar et al., 2015), WRKY45 (Wang et al., 2014), and WRKY75 in *Arabidopsis* (Devaiah et al., 2007a; Encinas-Villarejo et al., 2009), GbWRKY1 in cotton (Zhang et al., 2012) and OsWRKY74 in rice (Dai et al., 2016) function in response to LPS. The importance of WRKY transcription factors in the coordination of biological processes might reflect their flexible and diverse regulatory mechanisms, which are achieved by interactions with an array of proteins such as VQ proteins, MAPKs, chromatin remodeling proteins, calmodulin proteins, 14-3-3 proteins, WRKY or other transcription factors (Chi et al., 2013). However, the function of the majority of WRKY transcription factors in the plant LPS response and other biological processes has not been extensively characterized.

The 14-3-3 proteins are highly conserved eukaryotic proteins characterized by a conserved central core flanked by divergent regions at the N and C termini. These regions can be subdivided into two distinct groups: the *ε* group, and the plant-specific non-ε group. The 14-3-3 proteins act as molecular scaffolds or chaperones by physically interacting with target proteins *via* phosphorylated motifs containing phosphoserine residues of (R/K)XX(pS/pT)XP, (R/K)XXX(pS/pT)XP, and pS/pT-X1-2-COOH, in which pS and pT denote a phosphoserine and a phosphothreonine, respectively (Ormancey et al., 2017). The target proteins include H(+)-ATPase (Xu et al., 2016), nitrate reductase (Xu et al., 2016), ubiquitin ligase (Lu et al., 2016), protein kinases such as CDPKs (Ito et al., 2014), and transcription factors such as AtWRI1 (Kong and Ma, 2018), PIF7 (Huang et al., 2018), and bZIP (Takeo and Ito, 2017; Luang et al., 2018). The interaction of 14-3-3 proteins with other proteins alters the activity, stability, subcellular localization, or composition of the protein complex and this regulates physiological processes in plants that include metabolism, transport, growth, development, and stress responses (Ormancey et al., 2017). The expression of several 14-3-3 proteins is affected *in planta* in response to phosphorus deprivation (Cao et al., 2007) and these proteins have been implicated in plant responses to phosphorus deficiency (Cao et al., 2007; Ding et al., 2012; Xu et al., 2012a,b; Li et al., 2017; Yuan et al., 2017; Zhang et al., 2018). They function by modulating H+ efflux through affecting *Arabidopsis* plasma membrane H^+^-ATPase2 (AHA2) or AHA7 (Li et al., 2017), regulating leaf carbon allocation, increasing phloem sucrose transport to promote root growth, or activating root plasma membrane H(+)-ATPases to release more protons under phosphorus deficiency (Xu et al., 2012a,b). However, knowledge concerning the role of 14-3-3 proteins and the molecular basis for the plant response to phosphorus deficiency remain elusive.

Pepper (*Capsicum annuum* L.) is a member of the solanaceae and is an agriculturally important vegetable crop. It is mainly distributed or planted in uplands that contain soil-borne pathogens, including *Ralstonia solanacearum*, and that confront plants with other stresses such as LPS, which frequently cause growth retardation and yield loss (Forster et al., 1998). The application of phosphite, which causes phosphorus deficiency and growth retardation in pepper plants, significantly reduces the incidence of *Phytophthora* crown rot (Forster et al., 1998), indicates potential crosstalk between responses to pathogen attack and LPS. Notably, the WRKY protein CaWRKY58 was previously shown to negatively regulate the response of pepper to *Ralstonia solanacearum* (Wang et al., 2013). Here, we report that *CaWRKY58* is transcriptionally induced by LPS and positively regulates the response of pepper to LPS by directly targeting *PHR1* and physically interacting with 14-3-3.

## Materials and Methods

### Pepper and *N. benthamiana* Plant Cultivation and Phosphorus-Deficiency Treatment

Pepper (inbred line HN42) and *N. benthamiana* plants were cultivated as described previously (Cheng et al., 2017) before growth in hydroponic culture. The roots of plants at the seven-leaf stage were cleared and plants were transferred from soil to nutrient solution containing sufficient phosphorus (0.83 mM NH_4_^+^, 9 mM NO_3_^−^, 0.83 mM HPO_3_^2+^, 6.0 mM K^+^, 1.5 mM Ca^2+^, 0.75 Mm Mg^2+^, 0.75 mM SO_4_^2−^, 15.8 μM Fe^2+^, 10.3 μM Mn^2+^, 4.2 μM Zn^2+^, 43.5 μM B^+^, and 2.14 μM Cu^2+^). After 7 days, the plants were transferred to the above-mentioned hydroponic culture solution in which 0.83 mM HPO_3_^2+^ was replaced with 0.08 mM HPO_3_^2+^ to provide a LPS treatment. The plants were harvested at 7 dpt (days post treatment) and 30 dpt, to measure root system architecture (RSA) and root phosphorus content, respectively.

### Vector Construction

The full-length open reading frames (ORFs) of *CaWRKY58* and *Ca14-3-3* were cloned into the entry vector pDONR207 by BP reaction. After confirmation by sequencing, the ORFs were recombined into the destination vectors pEarleyGate103 [for C-terminal green fluorescent protein (GFP) fusions], pDEST-15 (for N-terminal GST fusions), pDEST17 (for N-terminal 6 × His fusions and expression in *Escherichia coli*) by LR reactions, using Gateway cloning (Invitrogen, Carlsbad, CA, United States). To construct vectors for VIGS-mediated gene silencing, a specific 300–400 bp fragment within the 3' UTRs of *CaWRKY58* or *CaPHR1* was amplified with specific primer pairs, using genomic DNA from pepper accession Zunla-1 as a template. The specificity of the amplicon was confirmed by BLAST search against the pepper genome.^1^ The PCR amplicon was cloned into pDONR207 by BP reaction and the identity of the clones was confirmed by sequencing before further subcloning into the PYL279 (pTRV2) vector, to generate *pTRV2:CaWRKY58*, or *pTRV2:CaPHR1*. All vectors were introduced into *Agrobacterium tumefaciens* strain GV3101.

### Virus-Induced Gene Silencing

*Agrobacterium tumefaciens* GV3101 cells containing *pTRV1*, *pTRV2:00*, *pTRV2:Ca* LPS, *pTRV2:CaWRKY58*, or *pTRV2:CaPHR1* were grown overnight in LB medium supplemented with appropriate antibiotics and were then pelleted and resuspended to a final cell density of OD_600_ = 0.8 in infiltration medium (10 mM MES, 10 mM MgCl_2_, 200 μM acetosyringone, and pH5.4). GV3101 cells harboring pTRV1 were mixed with cells containing *pTRV2:00*, *pTRV2:CaPDS*, *pTRV2:CaWRKY58* or *pTRV2:CaPHR1* in a 1:1 ratio and infiltrated into the cotyledons of 2-week-old pepper seedlings. The plants were then placed in a growth chamber at 16°C in the dark for 56 h, and were then transferred to a growth room at 25°C and 60% humidity, with a light intensity of 60–70 μmol photons m^−2^ s^−1^ and a 16-h light/8-h dark photoperiod, until the plants infiltrated with *pTRV:CaPDS* exhibited a bleached phenotype, indicative of successful silencing of the *PHYTOENE DESATURASE* gene. Around 36 pepper plants for each gene every time, and the experiment was replicated three times.

### Construction of Transgenic *N. benthamiana* Lines Overexpressing *CaWRKY58*

Transgenic *N. benthamiana* plants were generated by leaf transformation as described previously (Dang et al., 2013), using the plant expression vector *pEarleyGate103-CaWRKY58*. Transgenic lines (T_0_) were selected with 0.04% BASTA and 20 BASTA-resistant T_0_ plants were further confirmed by PCR and RT-PCR using a *CaWRKY58*-specific primer pair (Supplementary Table S1). Seeds of T_1_ lines were harvested from BASTA-resistant and self-pollinated T_0_ plants; similarly, seeds of T_2_ lines were harvested from BASTA-resistant and self-pollinated plants of T_1_ lines. Plants of T_2_ lines were used for future analysis after confirmation by RT-PCR using *CaWRKY58*-specific primer pair and western blotting using an anti-GFP antibody.

### Measurement of Total P and Pi Content in Roots of Pepper and *N. benthamiana*

Measurement of Pi concentration and plant total P content was performed as described previously (Nanamori et al., 2004; Zhou et al., 2008; Wu et al., 2011). The Pi concentration was normalized to fresh weight (FW) and the total P content was normalized to dry weight. Six biological replicates were performed in the experiment.

### Transient Expression of CaWRKY58 or Ca14-3-3

The plant expression vector *pEarleyGate103-CaWRKY58* (*Ca14-3-3*) was transformed into GV3101 cells, which were infiltrated into leaves of pepper or *N. benthamiana*, following the method described previously (Guan et al., 2018). Leaves were harvested at indicated time points for further experiments, including subcellular protein localization and total RNA extraction.

### Prokaryotic Expression of Fusion Proteins in *E. coli*

To purify soluble CaWRKY58-6 × His and Ca14-3-3-GST fusion proteins, a pDEST17 plasmid harboring the full-length ORF of *CaWRKY58* or the pDEST-15 vector containing the full-length ORF of *Ca14-3-3* was introduced into *E. coli* strain BL21. Expression of the fusion proteins was induced by the addition of 1 mM (final concentration) isopropyl *β*-D-1-thiogalactopyranoside (IPTG) at 20°C for 12 h. The presence of the soluble proteins in the supernatant of the *E. coli* BL21 cell lysate was confirmed by SDS-PAGE gel electrophoresis.

### Electrophoretic Mobility Shift Assay Analysis

Electrophoretic mobility shift assay (EMSA) was performed as described previously (Heravi and Altenbuchner, 2014; Shen et al., 2020), using CaWRKY58-6 × His purified from *E. coli* strain BL21 and the promoter fragments containing the wild-type or mutated W-box obtained from Cy5-labeled probes (*CaPHR1-P^cy5^*, *mCaPHR1-P^cy5^*) or Cy5-nonlabeled probes (CaPHR1-P; Supplementary Table S1). Before incubation with CaWRKY58-6 × His, CaPHR1-P^cy5^, and CaPHR1-P were mixed in different ratios to assay their competitive interaction with CaWRKY58-6 × His. The EMSA blot image was generated with Odyssey CLX (LI-COR). The experiment was replicated three times.

### Yeast Two-Hybrid Assay

The yeast two-hybrid assay was performed as described previously (Liu et al., 2015), the ORF of *CaWRKY58* was cloned into the *pDONR201* (satellite vector) and transferred into the *pDEST32* (destination vector) for the yeast two-hybrid screen. The yeast two-hybrid library screening was performed according to the manufacturer’s instructions (Invitrogen).

### Bimolecular Fluorescence Complementation Assay

The ORF of *CaWRKY58* and *Ca14-3-3* were cloned into the entry vector pDONR207 first, then the ORF of *CaWRKY58* was recombined into destination vector pEYFP-N1 (*CaWRKY58-YFP^N^*) and the ORF of *Ca14-3-3* was recombined into destination vector pEYFP-C1 (*Ca14-3-3-YFP^C^*). The constructs were transformed into the *Agrobacterium tumefaciens* strain GV3101, respectively. The GV3101 cells containing the two vectors were mixed in a 1:1 ratio and were infiltrated into *N. benthamiana* leaves. The cell layers of infiltrated leaves were visualized by microscopy (Leica, Germany) at 48 hours post infiltration (hpi). The experiment was replicated three times.

### Microscale Thermophoresis

The interaction between CaWRKY58 and Ca14-3-3 was confirmed by microscale thermophoresis (MST; Zillner et al., 2012). For this, Ca14-3-3-GFP, or GFP isolated by immunoprecipitation with an anti-GFP antibody from pepper leaves transiently overexpressing Ca14-3-3-GFP or GFP were used as targets, and CaWRKY58-6 × His was used as a ligand. Protein-protein interactions between Ca14-3-3-GFP or GFP, and CaWRKY58-6 × His, were measured using 20 nM Ca14-3-3-GFP or GFP. The CaWRKY58-6 × His solution was diluted to a concentration range from 1.0E^−10^ to 1.0E^−3^ mM and the CaWRKY58-6 × His fusion protein was incubated with the labeled protein for 10 min in interaction buffer. The samples were then loaded into Monolith NT.115 Capillaries (Cat. MO-K002, NanoTemper Technologies, Germany) using 50% IR laser power and an LED excitation source, with *λ* = 470 nm at ambient temperature. The *K_d_* values were calculated for interactions between Ca14-3-3-GFP and CaWRKY58-6 × His or GFP and CaWRKY58-6XHis using NanoTemper Analysis 1.2.20 software (Zillner et al., 2012; Papageorgiou et al., 2016). The experiment was replicated three times.

### Pull-Down Assay and Immunoblotting

The physical interaction between CaWRKY58 and Ca14-3-3 was confirmed by a pull-down assay. Ca14-3-3-GST fusion protein isolated from *E. coli* was incubated with GSH magnetic beads (Beaver, Suzhou, China) for 1 h, and CaWRKY58-GFP isolated from pepper leaves that transiently overexpressed *CaWRKY58-GFP* was added to the mixture and incubated for 1 h. After washing and elution, an appropriate amount of 1 M tris-HCl (pH 8.0) solution was added to adjust the pH. The protein was examined by immunoblotting with anti-GFP and anti-GST antibodies (Guan et al., 2018). The experiment was replicated three times.

### Quantitative Real-Time RT-PCR

A BIO-RAD Real-time PCR system (Foster City, CA, United States) and SYBR Premix Ex Taq II system (TaKaRa, Dalian, China) were used to quantify gene expression. Total RNA extraction and real-time RT-PCR were carried out as described previously (Dang et al., 2013; Cai et al., 2015; Guan et al., 2018). The specific primer pairs used are listed in Supplementary Table S1. The relative transcript level of each sample was normalized to that of *CaACTIN* (GQ339766). Six biological replicates were performed in the experiment.

### Chromatin Immunoprecipitation Analysis

Chromatin immunoprecipitation (ChIP) was performed as described previously (Guan et al., 2018). Briefly, CaWRKY58-GFP was transiently overexpressed in pepper leaves *via* agroinfiltration, and the DNA-protein complexes were isolated, sheared into fragments of 300–500 bp in length and immunoprecipitated using an anti-GFP antibody. The DNA was purified and used as a template for PCR and qPCR. The specific primer pairs of the fragment containing W-box in the promoter of *CaPHR1* and *CaNPR1* are listed in Supplementary Table S1. The experiment was replicated three times.

### *Ralstonia solanacearum* Infection

The *Ralstonia solanacearum* (FJC100301) cultivation and inoculation was performed as described previously (Dang et al., 2013; Shen et al., 2020). The pepper root was harvested at 24 h post inoculation for transcript level detection of related genes.

## Results

### *CaWRKY58* Is Upregulated by LPS in Pepper Plants

**Figure 1:**
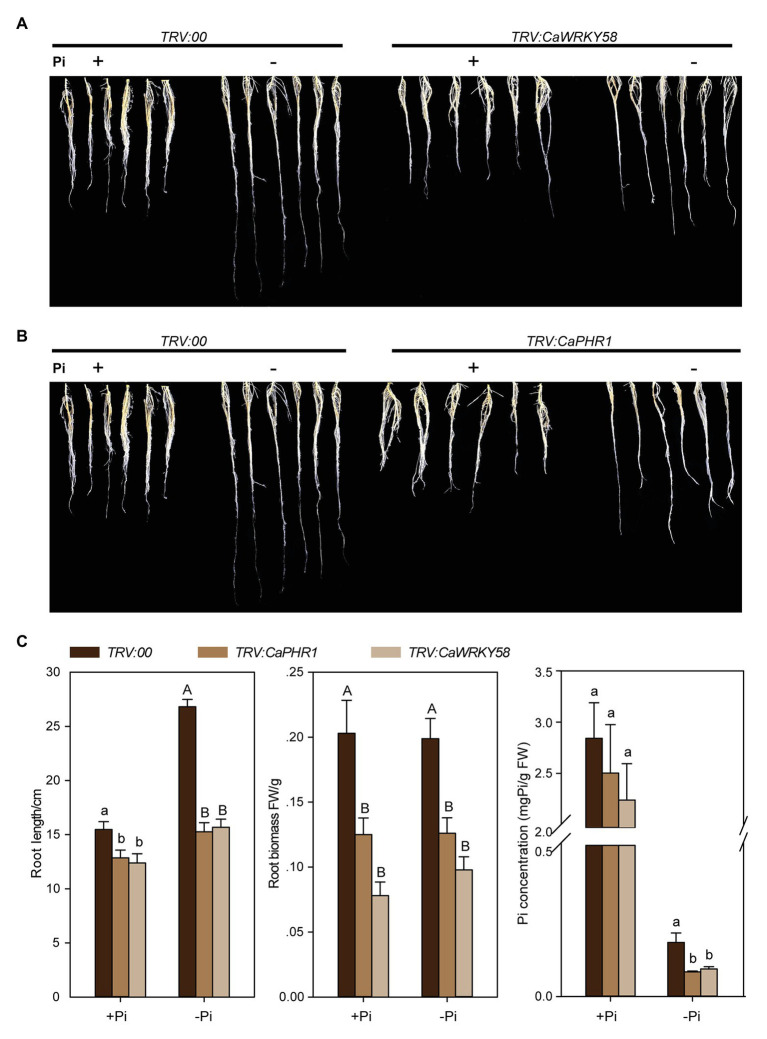
*CaWRKY58* is induced by and plays a role in pepper response to low-phosphorus stress (LPS). **(A)**, Relative transcript level of *CaWRKY58* in roots of pepper plants challenged with low-phosphorus stress LPS measured by quantitative real-time RT-PCR (qRT-PCR) at 1, 3, and 5 dpt (days post treatment). **(B)**, Root system architecture (RSA) of *Nicotiana benthamiana* plants overexpressing *CaWRKY58* following exposure to LPS. Transgenic *N. benthamiana* plants at the 4–6 leaf stage were grown in nutrient solution with sufficient Pi and were transferred to Pi-deficient solution for 7 days. Control plants were cultured in nutrient solution with sufficient Pi, (bars = 5 cm). **(C)**, Root length and weight of *N. benthamiana* plants overexpressing *CaWRKY58* following exposure to LPS. FW: fresh weight, WT: wild type, *CaWRKY58-OE*: *N. benthamiana* plants overexpressing *CaWRKY58*, +Pi: with sufficient Pi, −Pi: with low Pi. In **(A,C)**, the data represent the mean ± SD for three biological replicates. The uppercase and lowercase above the bars indicate significant differences (*p* < 0.01) and (*p* < 0.05), respectively, according to Fisher’s protected least significant difference (LSD) test.

*CaWRKY58* is constitutively expressed in pepper plants, but is downregulated by *Ralstonia solanacearum* infection (RSI) and negatively regulates the response to RSI (Wang et al., 2013). To test the potential role of *CaWRKY58* in other plant biological processes, we quantified the *CaWRKY58* transcript level in pepper plants challenged with several different stresses (salinity, heat stress, and drought), including LPS. Expression of *CaWRKY58* was upregulated by LPS at 3 and 5 dpt (Figure 1A), indicating that it might be involved in the LPS response.

### Ectopic Overexpression of *CaWRKY58* Significantly Enhanced the Tolerance of *Nicotiana benthamiana* to LPS

To further explore the role of *CaWRKY58* in the response to LPS in pepper, *N. benthamiana* plants were generated that transgenically overexpressed CaWRKY58. Twenty T_0_ transgenic plants were produced, and the corresponding T_1_ and T_2_ lines were produced. Three of the resulting lines with high levels of *CaWRKY58* expression were selected for further study. Transgenic and control plants were subjected to LPS, and their RSA and root Pi content were quantified. The three transgenic *N. benthamiana* lines exhibited an enlarged RSA and enhanced root Pi content compared with control plants (Figures 1B,C, Supplementary Figure S1), suggesting that overexpression of *CaWRKY58* enhanced the tolerance of *N. benthamiana* to LPS.

### Silencing *CaWRKY58* Compromised the Tolerance of Pepper Plants to LPS

**Figure 2:**
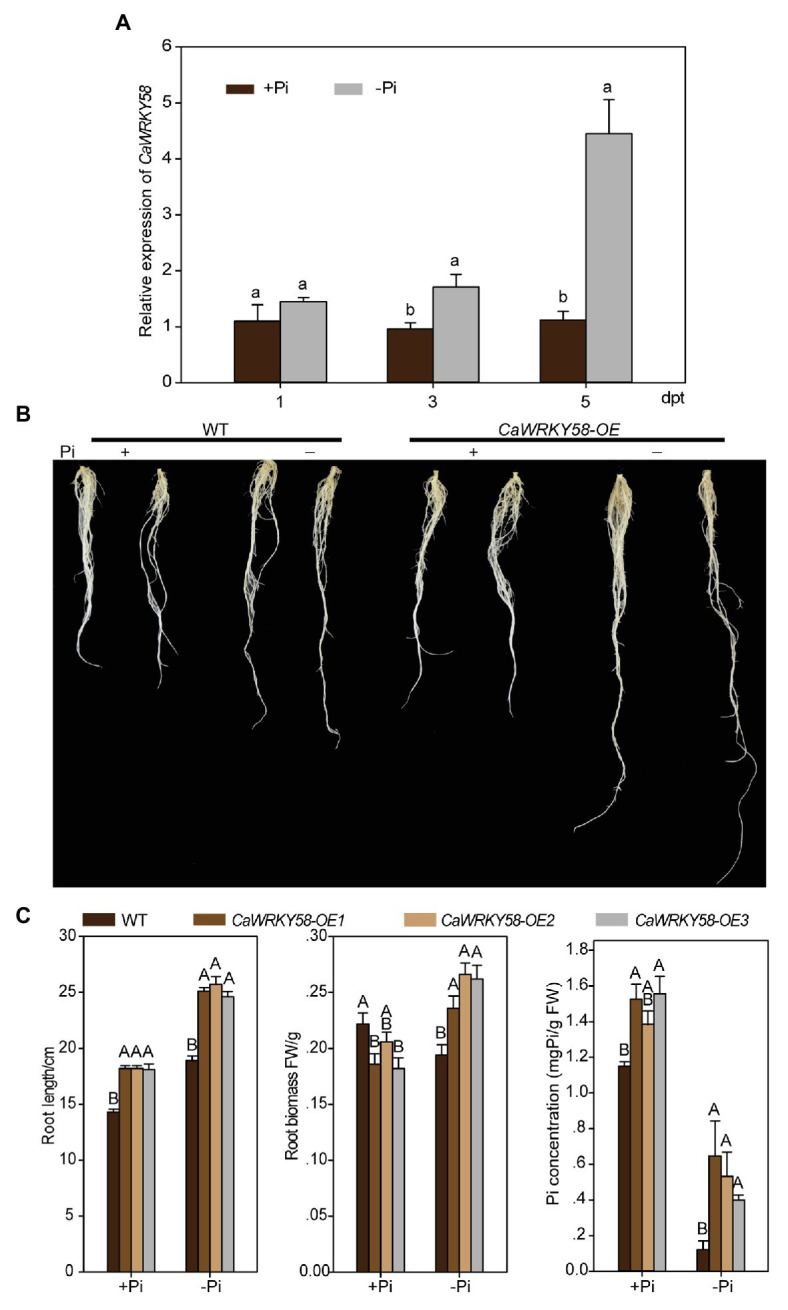
The effect of Pi deficiency on root growth of *CaPHR1* and *CaWRKY58*-silenced pepper plants. **(A)**, RSA of *CaWRKY58*-silenced pepper plants upon LPS. **(B)**, RSA of CaPHR1-silenced pepper plants treated with LPS at 7 dpt; both *CaWRKY58* and *CaPHR1* were silenced by virus-induced gene silencing (VIGS; bars = 3 cm). **(C)**, Root length and root weight of pepper plants subjected to LPS at 7 dpt. FW: fresh weight. In **(C)**, the data represent the mean ± SD for three biological replicates. The uppercase and lowercase above the bars indicate significant differences (*p* < 0.01) and (*p* < 0.05), respectively, according to Fisher’s protected LSD test.

To test the potential role of CaWRKY58 in the response of pepper plants to LPS, we generated *CaWRKY58*-silenced pepper plants *via* virus-induced gene silencing (VIGS) using a vector described previously (Wang et al., 2013). The efficiency and specificity of *CaWRKY58* silencing were assayed, and the transcript level of *CaWRKY58* in *TRV:CaWRKY58* pepper plants was approximately 20–30% that in control plants, the transcript level of *CaWRKY65*, whose sequence is more highly similar to that of *CaWRKY58* than other WRKY members in pepper, was not significantly affected by *CaWRKY58* silencing (Supplementary Figure S2), indicating that silencing was specific for *CaWRKY58*. The *CaWRKY58*-silenced pepper plants were subjected to LPS, and the RSA of *TRV:CaWRKY58* and wild-type control plants was enlarged upon LPS treatment compared with that in non-limiting phosphorus conditions, and the RSA of *TRV:CaWRKY58* plants under LPS was significantly reduced compared with that of control plants (Figures 2A,C). In parallel, the Pi content was analyzed and was significantly lower in *TRV:CaWRKY58* plants than in wild-type plants (Figure 2C). Collectively, these data indicate that CaWRKY58 positively regulates the response of pepper plants to LPS treatment.

### CaWRKY58 Directly Targets *CaPHR1* in Roots of Pepper Plants Under LPS

**Figure 3:**
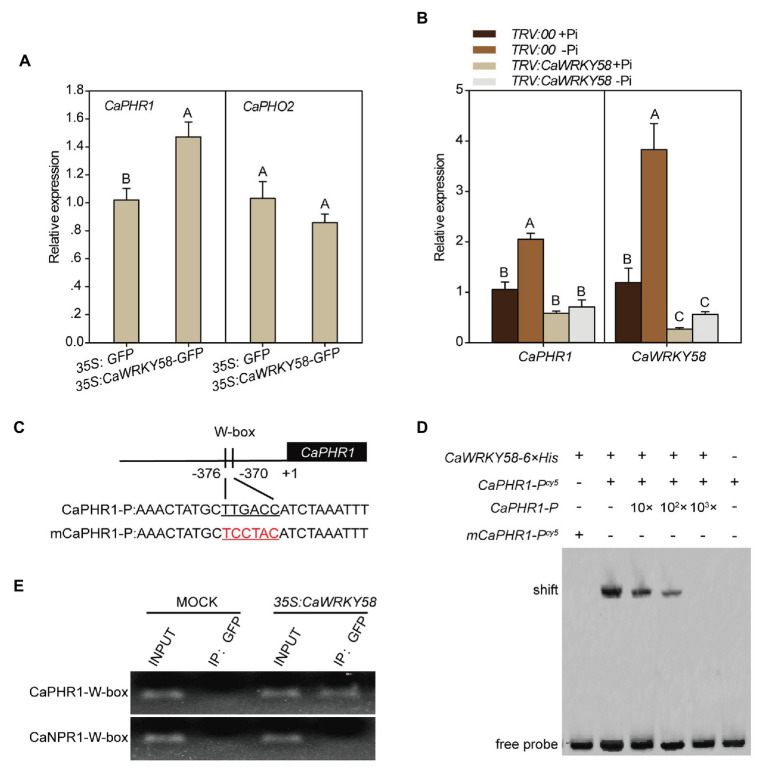
CaWRKY58 directly binds the promoter of *CaPHR1 via* the W-box and transcriptionally regulates its transcription. **(A)**, Effect of transient overexpression of *CaWRKY58-GFP* on the transcription of *CaPHR1* and *CaPHO2*. The relative transcript level of *CaPHR1* to that in the leaves of plants transiently overexpressing GFP was detected by RT-qPCR at 48 hours post infiltration (hpi). **(B)**, Effect of *CaWRKY58* silencing on the transcription of *CaPHR1* and *CaPHO2* in plants with and without LPS at 5 dpt. **(C)**, Location of the W-box in the promoter of *CaPHR1* and the sequence of the mutated W-box. **(D)**, Electrophoretic mobility shift assay (EMSA) for the binding of CaWRKY58-6 × His to the CaPHR1 promoter containing the W-box (CaPHR1-P) and to the promoter fragment of CaPHR1 containing a mutated W-box (mCaPHR1-P). **(E)**, Chromatin immunoprecipitation (ChIP)-qPCR assay showing that CaWRKY58 binds the W-box within the promoter of *CaPHR1*. CaWRKY58-GFP was transiently overexpressed in leaves for 48 h and the chromatin was isolated, sheared into 300–500 bp fragments and immunoprecipitated using an anti-GFP antibody. The purified DNA fragments were used as a template for qPCR with a specific primer pair to the promoter fragment of *CaNPR1* and *CaPHR1* containing the W-box. In **(A,B)**, the data represent the mean ± SD for three biological replicates. The uppercase above the bars indicates a significant difference (*p* < 0.01) according to Fisher’s protected LSD test.

Given that WRKY proteins function mainly by binding the conserved W-box in promoters of their target genes, and CaWRKY58 regulates the response of pepper plants to LPS, we hypothesized that CaWRKY58 positively regulates the response to LPS by binding to the W-box in the promoters of genes related to LPS tolerance. To identify the potential target genes of CaWRKY58 in response to LPS, the promoters of potential LPS-responsive genes were screened for the presence of a W-box, which was identified in the promoter of *CaPHR1* (LOC107863907) in the pepper genome. CaPHR1 is the ortholog of PHR1, and PHR1 is a MYB transcription factor that positively regulates the response of different plant species to LPS (Nilsson et al., 2007; Valdes-Lopez et al., 2008a; Ren et al., 2012; Ruan et al., 2015; Pacak et al., 2016; Xue et al., 2017). To assess whether *CaPHR1* is regulated by CaWRKY58 in response to LPS, the level of *CaPHR1* expression was quantified in the leaves of pepper plants that transiently overexpressed *CaWRKY58* and was significantly higher than that in the leaves of control plants. However, the expression of *CaPHO2* (the ortholog of *PHOSPHATE2* in pepper) was not significantly different to that in control plants (Figure 3A). By contrast, LPS significantly upregulated *CaPHR1*, whereas silencing *CaWRKY58* by VIGS significantly blocked this upregulation in leaves (Figure 3B). To elucidate the role of CaPHR1 in the response to LPS, *CaPHR1* was silenced by VIGS and the silenced plants were subjected to LPS. A reduced RSA was observed in *TRV:CaPHR1* plants compared to in the wild-type plants at 7 dpt, which was similar to that in *TRV:CaWRKY58* plants (Figures 2B,C). These results indicate that CaWRKY58 positively regulates the response to LPS by regulating the expression of *CaPHR1*.

To analyze whether *CaPHR1* is directly targeted by CaWRKY58, the ability of CaWRKY58 to bind the *CaPHR1* promoter was studied by EMSA using CaWRKY58-6 × His purified from *E. coli* and the promoter of *CaPHR1* (*CaPHR1-P^cy5^*), which contains a W-box. We detected a mobility shift when CaWRKY58-6 × His was incubated with excess *CaPHR1-P* (*CaPHR1-P^cy5^*), but no mobility shift was detected when CaWRKY58-6 × His was incubated with excess *CaPHR1-P* containing a mutated W-box (*mCaPHR1-P^cy5^*; Figures 3C,D). In parallel, a ChIP-PCR assay showed that CaWRKY58-GFP was enriched at the W-box containing *CaPHR1* promoter but not at the W-box containing *CaNPR1* promoter (*mCaPHR1-P*; Figure 3E). These results indicate that CaWRKY58 directly bind to the promoter of *CaPHR1*.

### Ca14-3-3 Interacts With CaWRKY58

**Figure 4:**
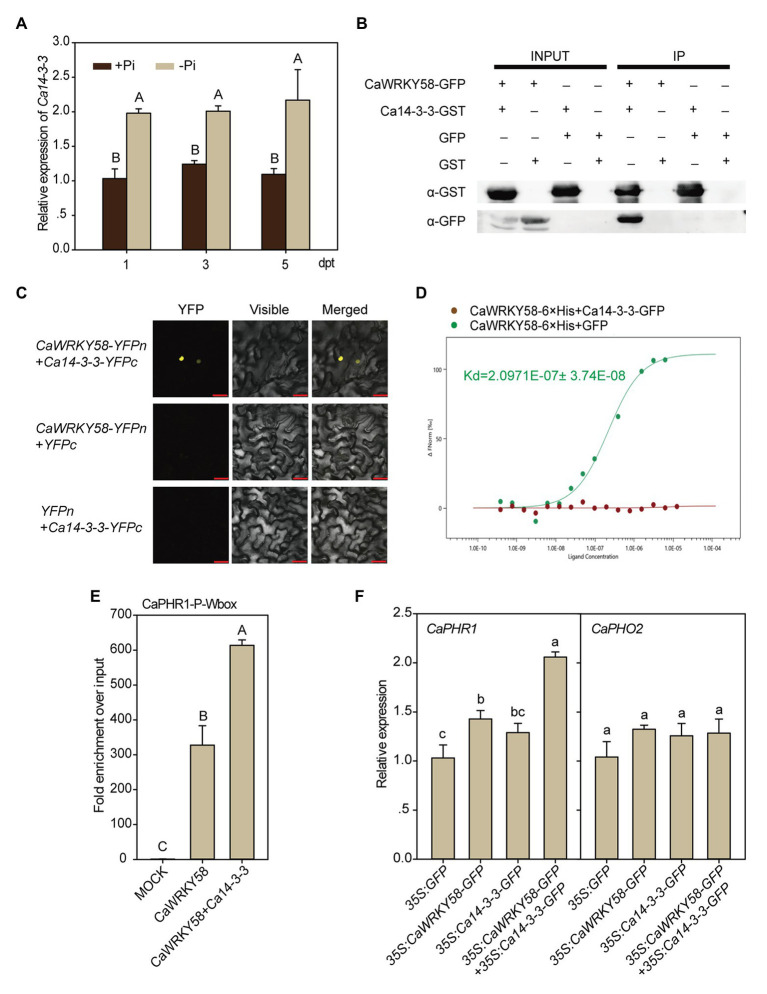
The transcriptional activation of *CaPHR1* by CaWRKY58 is enhanced by interaction between CaWRKY58 and Ca14-3-3. **(A)**, Transcription of *Ca14-3-3* was induced by LPS in roots of pepper plants. **(B)**, Pull-down assay to show the interaction between Ca14-3-3 and CaWRKY58. CaWRKY58-GFP was transiently overexpressed in leaves of pepper plants by agroinfiltration. Ca14-3-3-GST fusion protein purified from *Escherichia coli* (BL21) by GST magnetic beads was used as the bait protein, the protein complex was further purified using GST magnetic beads, and CaWRKY58 in the protein complex was detected by immunoblotting with an anti-GFP antibody. **(C)**, Interaction between Ca14-3-3 and CaWRKY58 assayed by bimolecular fluorescence complementation (BiFC; bars = 25 μM). **(D)**, Ca14-3-3/CaWRKY58 interaction was assayed by microscale thermophoresis (MST), CaWRKY58-6 × His (as the ligand) was purified from *E. coli* (BL21) and Ca14-3-3-GFP (as the target) was transiently overexpressed in leaves of pepper plants by agroinfiltration. **(E)**, Effect of transient overexpression of Ca14-3-3 on the binding of CaWRKY58 to the *CaPHR1* promoter by ChIP-qPCR assay. **(F)**, Effect of transient overexpression of *Ca14-3-3* on *CaPHR1* and *CaPHO2* transcription with or without *CaWRKY58* transient co-overexpression. In **(A,E,F)**, the data represent the mean ± SD for three biological replicates. The uppercase and lowercase above the bars indicate significant differences (*p* < 0.01) and (*p* < 0.05), respectively, according to Fisher’s protected LSD test.

The function of WRKY transcription factors is often modulated by other regulatory proteins *via* protein-protein interactions (Chi et al., 2013). To identify the potential interacting partners of CaWRKY58, a yeast two-hybrid assay was performed using CaWRKY58 as a bait. Among the 25 positive clones, a 14-3-3 protein (LOC107867389) was identified. Because the deduced amino-acid sequence contained a conserved 14-3-3 domain and exhibited a high sequence identity to 14-3-3 proteins (Supplementary Figure S3), we named this protein Ca14-3-3. The expression level of *Ca14-3-3* was significantly enhanced by LPS treatment (Figure 4A). The interaction between CaWRKY58 and Ca14-3-3 was confirmed by a pull-down assay by incubating Ca14-3-3-GST purified from *E. coli* with proteins isolated from pepper leaves that transiently overexpressed CaWRKY58-GFP. Following purification with GST magnetic beads, the protein complex that included Ca14-3-3-GST was immunoblotted with an anti-GFP antibody, which detected the presence of CaWRKY58-GFP. As expected, this demonstrated that Ca14-3-3 interacted with CaWRKY58 (Figure 4B). In parallel, the interaction between 14-3-3 and CaWRKY58 was further confirmed by bimolecular fluorescence complementation (BiFC) in *N. benthamiana* leaves. The leaves were analyzed at 48 hpi and YFP fluorescence was detected in the nucleus of epidermal cells of leaves co-expressing CaWRKY58 and Ca14-3-3 fusion proteins, but no fluorescence was observed in control leaves (Figure 4C). In addition, we purified CaWRKY58-6 × His from *E. coli* soluble extracts. Purified CaWRKY58-6 × His fusion protein was serially diluted and mixed with a constant amount of Ca14-3-3-GFP before performing an MST assay, which allowed the dissociation constant (*K_d_*) of the CaWRKY58-6 × His-Ca14-3-3-GFP complex to be calculated. The CaWRKY58-Ca14-3-3 protein pair produced a clear binding curve, with a *K_d_* of 2.0971E^−7^ M (Figure 4D). Collectively, these data indicate that CaWRKY58 and Ca14-3-3 physically interact with each other.

### The Transcriptional Activation of *CaPHR1* by CaWRKY58 Is Enhanced by Interaction Between CaWRKY58 and Ca14-3-3

Because CaWRKY58 interacts with Ca14-3-3 and directly transcriptionally upregulates *CaPHR1* during the response of pepper to LPS, we hypothesized that Ca14-3-3 might affect the function of CaWRKY58 in this process. To test this hypothesis, we analyzed the effect of transient co-overexpression of CaWRKY58 and Ca14-3-3 in leaves of pepper plants on the transcription of *CaPHR1*, by qRT-PCR. The transcript level of *CaPHR1* was promoted by transient overexpression of *CaWRKY58* alone, and this promotion was enhanced by the additional transient overexpression of Ca14-3-3, although the overexpression of Ca14-3-3 alone did not significantly enhance the transcription of *CaPHR1*. By contrast, the expression of *CaPHO2* was not affected by either transient overexpression of *CaWRKY58* or its transient overexpression together with *Ca14-3-3* (Figure 4F). To test whether binding of the *CaPHR1* promoter by CaWRKY58 was enhanced by the interaction of Ca14-3-3 with CaWRKY58; ChIP-qPCR assay was performed on chromatin isolated from leaves of pepper plants that either transiently overexpressed *CaWRKY58-GFP* alone, or *CaWRKY58-GFP* and *Ca14-3-3-GFP* together. The chromatin was sheared into fragments 300–500 bp in length and was immunoprecipitated with an anti-GFP antibody, and DNA derived from the immunoprecipitated chromatin was used as template for ChIP-qPCR using a primer pair specific for the W-box within the *CaPHR1* promoter. The W-box sequence was significantly enriched by transient co-overexpression of *Ca14-3-3-FLAG* and *CaWRKY58-GFP* (Figure 4E). Consistent with this, the transcript level of *CaPHR1* in plants that transiently co-expressed *CaWRKY58-GFP* and *Ca14-3-3-GFP* was significantly higher than that in plants that expressed *CaWRKY58-GFP* or *Ca14-3-3-GFP* alone (Figure 4F). These data indicate that the transcriptional regulation of *CaPHR1* by CaWRKY58 is enhanced by Ca14-3-3.

### Genes Related to Immunity or LPS Tolerance Are Regulated by CaWRKY58 in a Context-Dependent Manner

Previous study showed that CaWRKY58 negatively regulates the response of pepper to RSI by downregulating immunity-related genes, including *CaDEF1* and *CaNPR1* (Wang et al., 2013). Here, we demonstrate that CaWRKY58 positively regulates the response to LPS by upregulating *CaPHR1*. To address whether CaWRKY58 regulates its target genes in a context-dependent manner, the transcript levels of *CaPHR1*, *CaNPR1*, and *CaDEF1* were quantified in pepper plants challenged with LPS and RSI. The expression of *CaPHR1* was only upregulated by LPS, whereas that of *CaDEF1* and *CaNPR1* was upregulated by RSI, indicating that the expression of these genes is context dependent (Supplementary Figure S4). Furthermore, the transcript levels of *CaDEF1*, and *CaNPR1* in *CaWRKY58*-silenced pepper plants were only enhanced in the presence of sufficient Pi, but not under LPS (Supplementary Figure S5). Furthermore, although the positive transcriptional regulation of *CaPHR1* by CaWRKY58 was enhanced by transient co-overexpression of Ca14-3-3, the transcriptional regulation of *CaDEF1* and *CaNPR1* by CaWRKY58 was not affected by Ca14-3-3 (Supplementary Figure S6). These data indicate that the activation of *CaPHR1* expression by CaWRKY58 occurs only in LPS but not in RSI; on the other hand, the negative regulation of *CaDEF1* and *CaNPR1* by CaWRKY58 does not occur under LPS.

## Discussion

Although WKRY and 14-3-3 proteins have been previously been shown to participate in plant immunity and the LPS response, and some members of both families physically interact, their functions in crosstalk between plant immunity and response to LPS remain uninvestigated. In this study, we provide evidence that in addition to acting as a negative regulator of immunity against RSI in pepper, CaWRKY58 positively regulates tolerance to LPS, an effect that is mediated by interaction with Ca14-3-3.

### CaWRKY58 Positively Regulates the Response to LPS by Directly Regulating *CaPHR1*

The data from CaWRKY58 upregulation against LPS and the data from loss--of-function and gain-of-function assay indicate that CaWRKY58 positively regulates the response to LPS by regulating *CaPHR1*, which related to LPS tolerance (Motte and Beeckman, 2017; Wang et al., 2019), as well as by modulating RSA, a key parameter that is positively related to LPS tolerance (Jain et al., 2007; Mori et al., 2016). Primary root growth was strongly inhibited by LPS in *Arabidopsis* (Peret et al., 2011; Gutierrez-Alanis et al., 2018); however, we observed that the growth of primary and lateral roots in pepper was promoted by P deficiency, which was enhanced by *CaWRKY58* overexpression (Figure 1B). This indicates that the regulation of RSA by LPS might differ among plant species. The data also indicate that CaWRKY58 positively regulates *CaPHR1* expression directly, because the W-box within the *CaPHR1* promoter was bound by CaWRKY58 according to ChIP-qPCR and EMSA (Figure 3D). PHR1 is a MYB-CC-type transcription factor that plays a key role in regulating the expression of Pi starvation-induced (PSI) genes, which leads to enhanced phosphate uptake (Nilsson et al., 2007). We conclude that CaWRKY58 positively regulates the LPS response in pepper by targeting *PHR1*.

### Ca14-3-3 Promotes the Transcriptional Activation Activity of *CaWRKY58* During LPS

In addition to regulation at the transcriptional level, the expression and function of transcription factors is frequently regulated post-translationally *via* interactions with other proteins (Schutze et al., 2008; Chi et al., 2013; Alves et al., 2014; Pireyre and Burow, 2015; Ohama et al., 2017). In particular, WRKY transcription factors interact with other WRKY proteins, VQ motif-containing proteins, MAPKs, chromatin remodeling proteins, calmodulin, and 14-3-3 proteins (Chi et al., 2013). 14-3-3 proteins regulate transcription by interacting with the transcription factors or activators RSG (Igarashi et al., 2001; Ishida et al., 2008), GmMYB176 (Li et al., 2012), MYBS2 (Chen et al., 2019), and CBF (Liu et al., 2017), and modifying their subcellular localization, stability, or transcription, leading to an appropriate transcriptional output. Moreover, 14-3-3 proteins have been implicated in the response to P deficiency (Wang et al., 2002; Xu et al., 2012b); however, it remains unknown whether 14-3-3 proteins function as transcription activators in these conditions. The data here indicate that the transcriptional activation of *CaPHR1* by CaWRKY58 is enhanced by the interaction between CaWRKY58 and Ca14-3-3, suggesting that Ca14-3-3 might promote the transcriptional activation activity of CaWRKY58 during the P-deficiency response. Because the interaction between 14-3-3 proteins and their protein partners is either dependent on or related to phosphorylation (Li et al., 2012; Ito et al., 2014; Chen et al., 2019) mediated by kinases including CDPKs (Ishida et al., 2008; Liu et al., 2017), we speculate that the interaction between Ca14-3-3 and CaWRKY58 might be associated with phosphorylation mediated by unidentified kinases. The further identification of these kinases might provide insight into the mechanism that underlies the LPS response in pepper.

### CaWRKY58 Functions in LPS and Immune Signaling, and Is Specifically Activated in the LPS Response by Ca14-3-3

We demonstrated previously that CaWRKY58 negatively regulates the response to RSI in pepper (Wang et al., 2013); therefore, CaWRKY58 functions in the response of pepper to both RSI and LPS. These two stresses activate convergent signaling nodes, such as WRKY45 (Shimono et al., 2007; Wang et al., 2014), WRKY75 (Devaiah et al., 2007a; Encinas-Villarejo et al., 2009), and PR10a (Huang et al., 2016), suggesting that plant immunity might be closely related to phosphorus nutrition. On the one hand, application of sufficient phosphate fertilizer is generally essential to increase root ramification and strength, thereby conferring vitality and disease resistance to plants (Sharma et al., 2013). By contrast, phosphate starvation can result in the repression of plant immunity (Finkel et al., 2019), and in *Arabidopsis*, PHR1 also directly represses immune responses (Castrillo et al., 2017). On the other hand, pathogen infection might decrease phosphate availability (Petters et al., 2002). However, the significance of these shared regulatory proteins in plant adaptation to the environment and their contribution to the relationship between plant phosphorus nutrition and disease resistance remains to be elucidated. The data here showed that LPS tolerance and immunity-related genes were differentially activated in pepper plants upon RSI and LPS treatments, and immunity-related genes, including *CaDEF1* and *CaNPR1*, were not negatively regulated by CaWARKY58 in plants exposed to LPS treatment, or following transient co-overexpression of CaWRKY58 and Ca14-3-3. This indicates that CaWRKY58 and Ca14-3-3 contribute to the positive regulation of CaWRKY58 in response to LPS, but do not contribute to its negative regulation in immunity against RSI.

Collectively, the data in this study show that CaWRKY58 acts as positive regulator of LPS in pepper plants by directly targeting and regulating *CaPHR1* and Ca14-3-3 acts as a specific transcriptional activator of CaWRKY58 during the LPS response.

## Data Availability Statement

The original contributions presented in the study are included in the article/Supplementary Material and further inquiries can be directed to the corresponding author.

## Author Contributions

SH and JC conceived the research and designed the experiments. JC, XH, SY, WC, JW, XX, FY, and YS performed the experiments. JC, SY, and DG analyzed the data. SH wrote the manuscript. All authors contributed to the article and approved the submitted version.

### Conflict of Interest

The authors declare that the research was conducted in the absence of any commercial or financial relationships that could be construed as a potential conflict of interest.

## Footnotes

^1^http://peppersequence.genomics.cn/page/species/blast.jsp
