# Supplementary material for: Ca14-3-3 Interacts With CaWRKY58 to Positively Modulate Pepper Response to Low-Phosphorus Starvation
Source: Front Plant Sci. 2021 Jan 14;11:607878. doi: 10.3389/fpls.2020.607878 (PMC7840522; doi:10.3389/fpls.2020.607878)
Supplement: Supplementary file 1 [file Table_1.docx]

Table S1. Primers used in this study.

| Name | Forward primer | Reverse primer |
| --- | --- | --- |
| Primers for full‐length gene cloning | | |
| *CaWRKY58* | ATGAATTTGACGTTGGCTCA | TCAACGCCAAGTATCTTTGG |
| *Ca14-3-3* | ATGGCGGCGGCGGCGGCAAT | TCAGGATTCATCCAACTGAT |
| Primers for gene silencing | | |
| *CaWRKY58* | TCTGCTTTGGATAGTACAGACGG | AGCTACTGTGCCATGTGATGA |
| *CaPHR1* | AAGTGATTGGCAGGAATGGGC | CGCATGCGTTGCTTTGTTGC |
| Primers to detect relative expression levels | | |
| *CaWRKY58* | AGCTCGGATGGGTCTTCTCC | AGCCTCAACTGCCATCTTCG |
| *Ca14-3-3* | GGATGGGCTCTAAATGTAGTTTGC | GGCAGCGACTGTTACCAAAG |
| *CaPHR1* | GCAAGTCAATGCCTGGTGTG | AGAACTTCCCTTTCCCGCAG |
| *CaPHO2* | CCACCCAAGCATTTTGAGGC | TGGTCCTGTTTCTTGGGTTGA |
| Primers for the segment containing W-box in the *CaPHR1* promoter | | |
| CaPHR1-Wbox | CATTTGAGGGTTTAGCATG | CAACAAGCATGCTACAACTG |


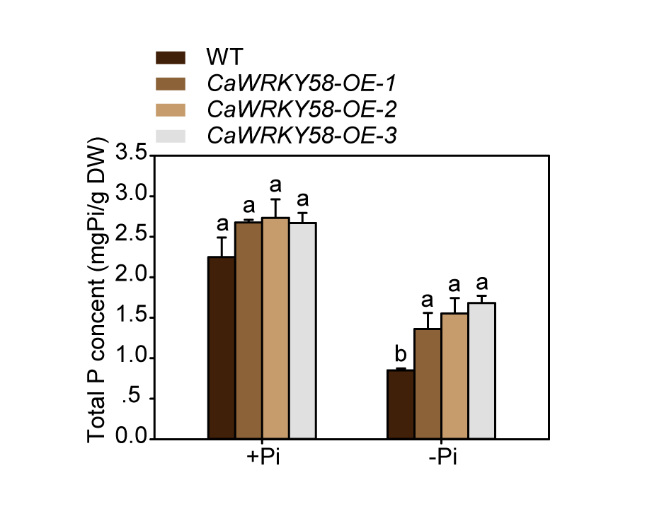


Figure S1. Total P concentration in roots of *Nicotiana benthamiana* plants overexpressing *CaWRKY58* upon LPS treatment at 20 dpt. Data represent the mean ± SD for three biological replicates. Lowercase letters above the bars indicate significant differences (*P* < 0.05) according to Fisher’s protected LSD test.


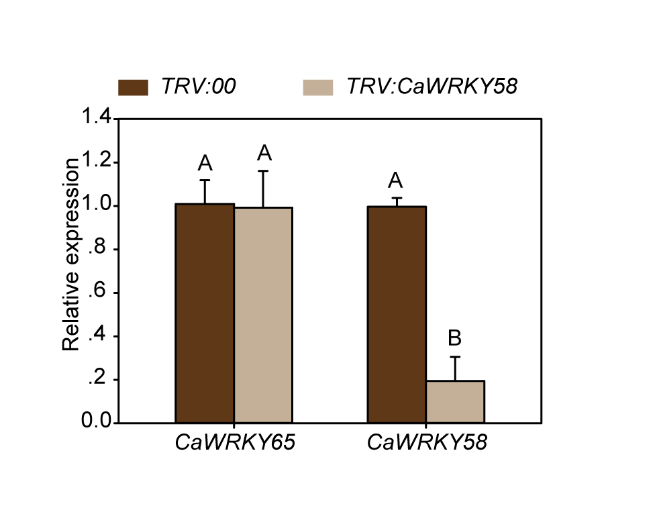


Figure S2. Success and specificity of *CaWRKY48* silencing displayed by relative transcript levels of *CaWRKY58* and *CaWRKY65* in leaves of *CaWRKY58*-silenced pepper plants compared to that of *CaWRKY65* in mock-treated pepper leaves.


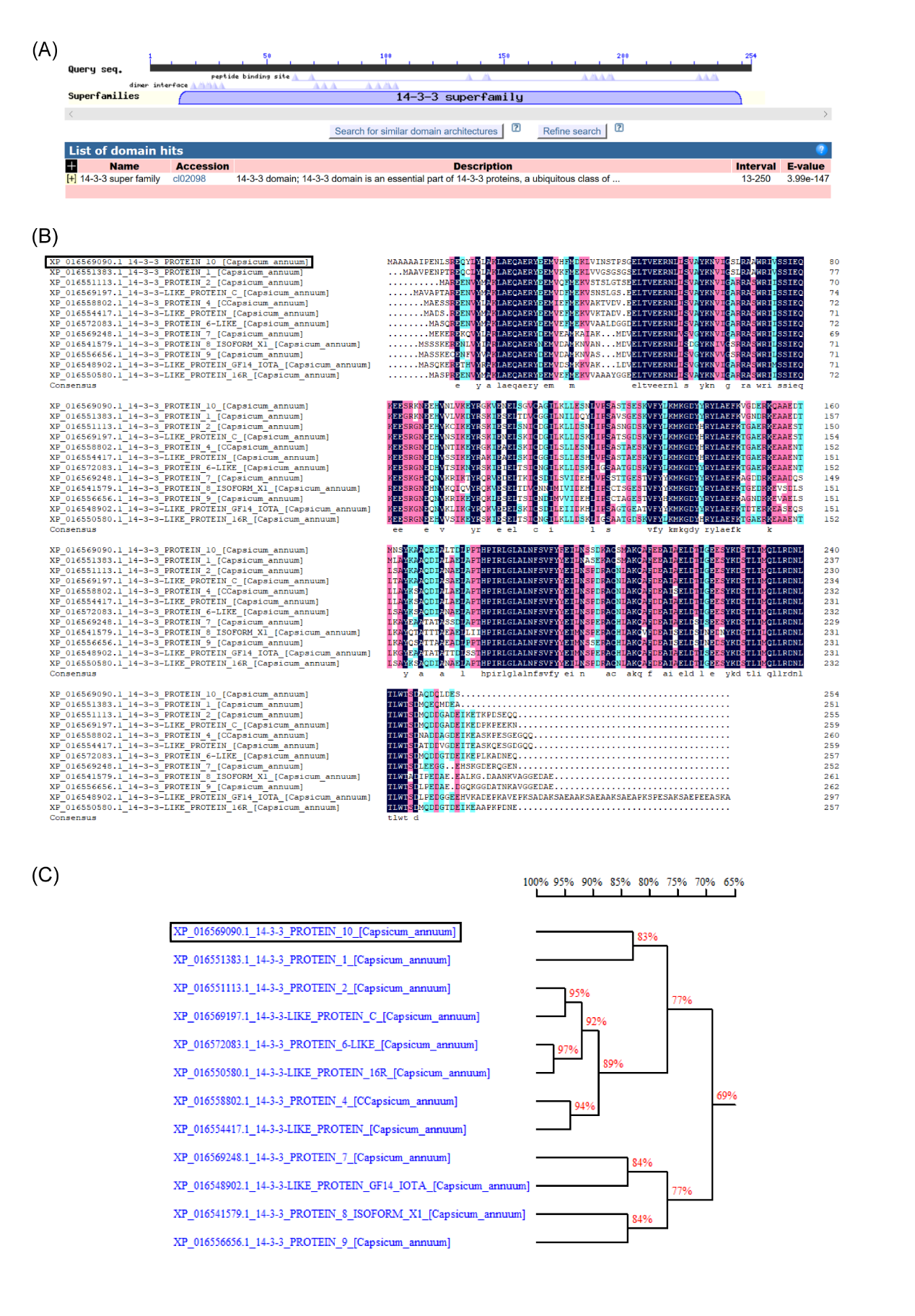


Figure S3. Pepper Ca14-3-3 and its putative orthologs.

(A), The conserved domain present in Ca14-3-3 (<https://www.ncbi.nlm.nih.gov/cdd>). (B), Comparison of the deduced amino-acid sequences of Ca14-3-3 and its putative orthologs. Sequences were aligned in DNAMAN using default parameters. Green shading, 50%–75% identity; red shading, 75%–100% identity, black shading, 100% identity. (C), Phylogenetic analysis of Ca14-3-3 and its putative orthologs.


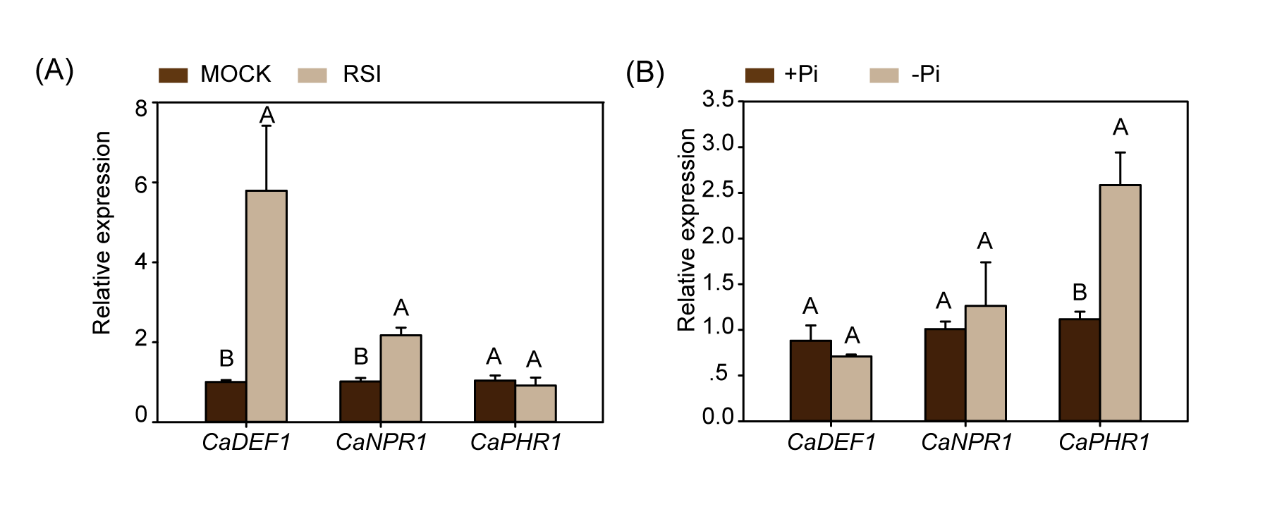


Figure S4. Effect of low-phosphorus stress (LPS) or *Ralstonia solanacearum* inoculation (RSI) on the transcription of genes related to LPS tolerance and immunity at 3 dpt and 12 hpi, respectively. +Pi: Supply with sufficient phosphorus, −Pi: treated with low-phosphorus stress. The data represent the mean ± SD for three biological replicates. Uppercase above the bars indicate significant differences (*P* < 0.01) according to Fisher’s protected LSD test.


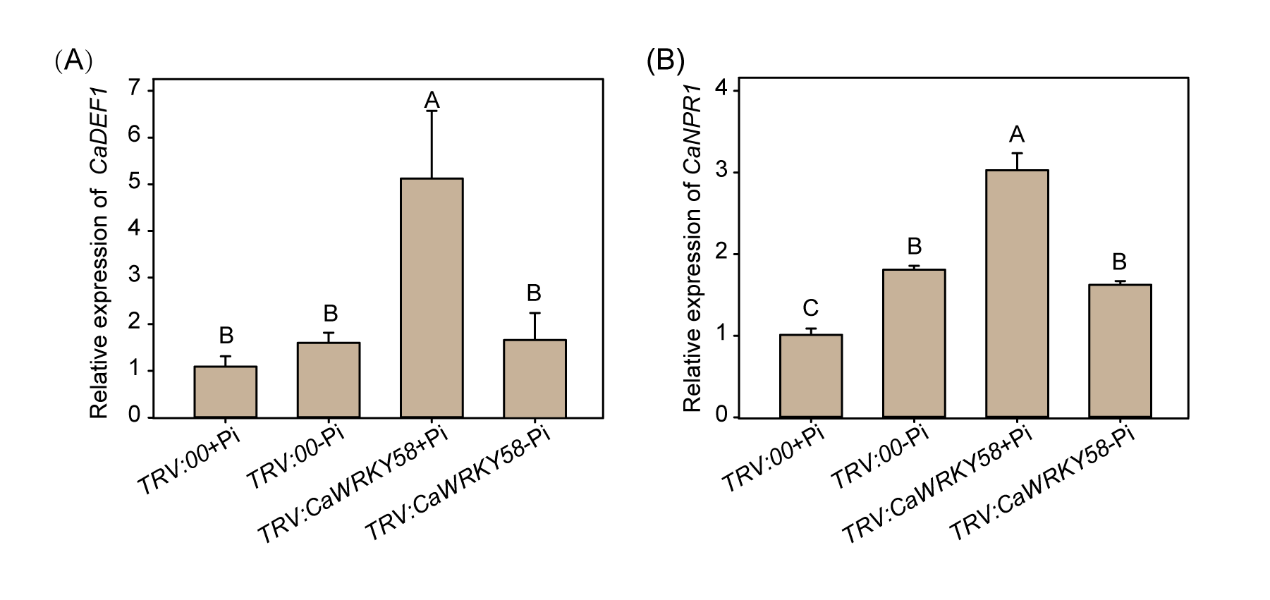


Figure S5. Effect of silenced *CaWRKY58* on the transcription of the immunity-related genes *CaDEF1* and *CaNPR1* in pepper plants with or without low-phosphorus stress. +Pi: sufficient phosphorus supply; −Pi: low-phosphorus stress treatment. The data represent the mean ± SD for three biological replicates. Uppercase above the bars indicate significant differences (*P* < 0.01) according to Fisher’s protected LSD test.


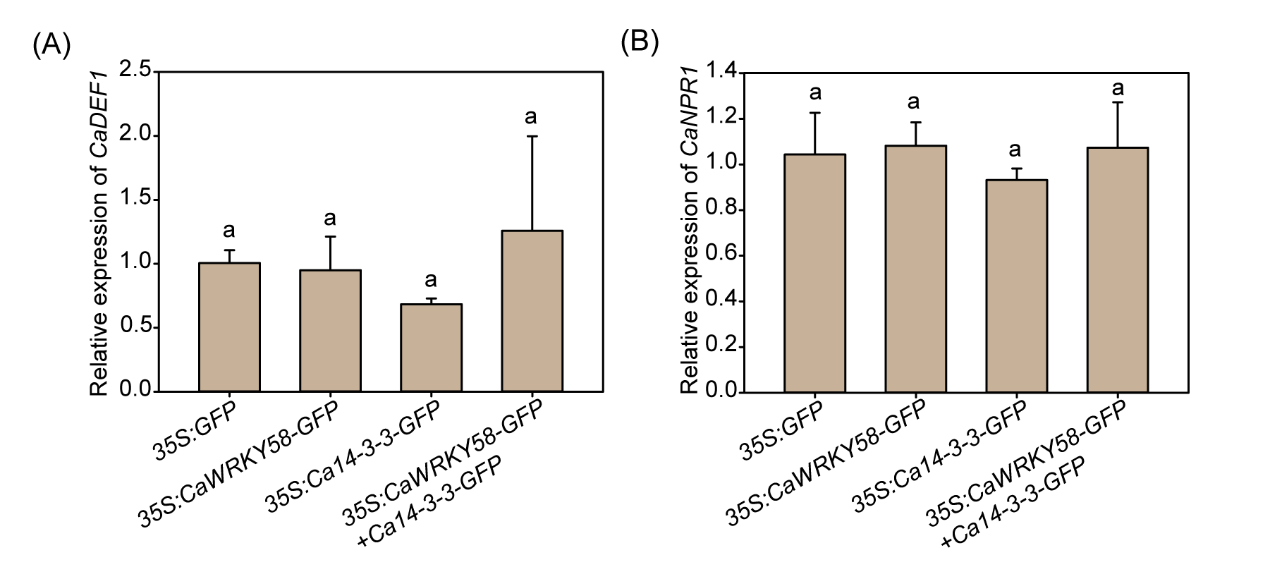


Figure S6. Effect of transient overexpression of *CaWRKY58* or *Ca14-3-3* alone, or *CaWRKY58* and *Ca14-3-3* together, on expression of the immunity-related *CaDEF1* and *CaNPR1* genes in pepper leaves. The data represent the mean ± SD for three biological replicates. Significant differences were by analyzed according to Fisher’s protected LSD test.
